# Supplementary material for: ARP-T1-associated Bazex–Dupré–Christol syndrome is an inherited basal cell cancer with ciliary defects characteristic of ciliopathies
Source: Commun Biol. 2021 May 10;4:544. doi: 10.1038/s42003-021-02054-9 (PMC8110579; doi:10.1038/s42003-021-02054-9)
Supplement: Supplementary file 1 — Supplementary Information [file 42003_2021_2054_MOESM1_ESM.pdf]

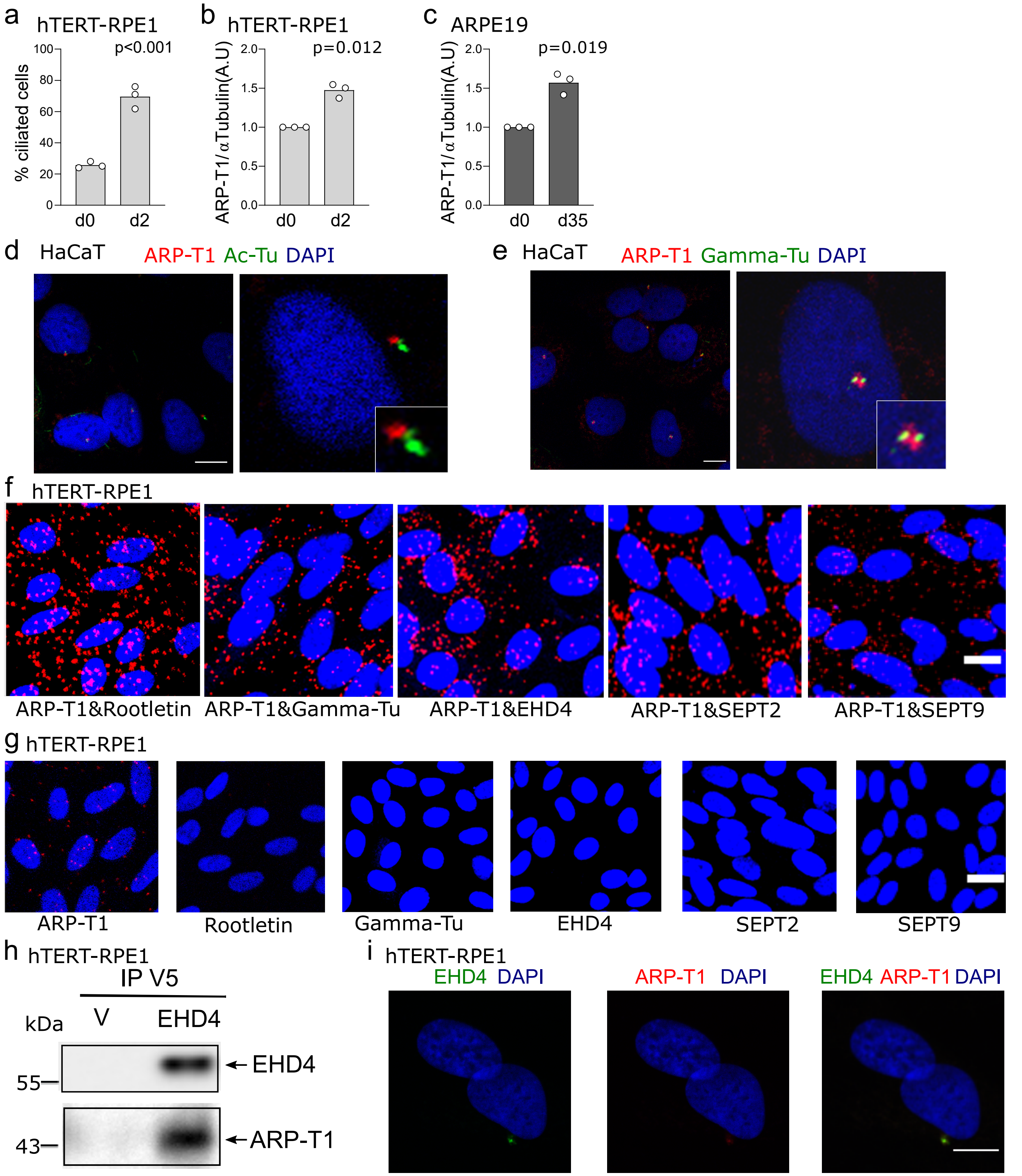

Supplementary Fig.1. Ciliogenesis and ARP-T1 increase after differentiation, and ARP-T1 localization and interactions.

a, Percentage of ciliated hTERT-RPE1 cells under proliferating (d0) and differentiating (d2) conditions. Data are presented as means of the percentage. Each open circle represents one independent experiment (N=3). **b**,**c**, Quantification of ARP-T1 expression in hTERT-RPE1 (**b**) and ARPE19 (**c**). Data are presented as means of the fold change compared to the value of undifferentiated samples. Each open circle represents one independent experiment (N=3). **d**,**e**, Immunofluorescence stainings of ARP-T1 (red) and acetylated-tubulin (green) (**d**), and ARP-T1 (red) and gamma-tubulin (green) (**e**) in 7 days differentiated HaCaT cells. Nuclei are stained with DAPI (blue). Scale bar, 10 µm. **f**, Proximity-mediated ligation assays using ARP-T1 and rootletin, or gamma-tubulin, or EHD4, or septin 2, or septin 9 antibodies, in 48 h serum-starved hTERT-RPE1 cells.
g, Proximity-mediated ligation assays using ARP-T1, rootletin, gamma-tubulin, EHD4, septin 2, or septin 9 antibody alone, in 48h serum-starved hTERT-RPE1 cells. **f**,**g**, Interactions are in red, nuclei are stained with DAPI (blue). Scale bar, 20 µm.
h, hTERT-RPE1 cells were transfected with EHD4 and empty vector, serum-starved for 48 h, and immunoprecipitated (IP) with anti-V5 antibody-conjugated agarose, and analyzed by immunoblot with indicated antibodies. **i**, Immunofluorescence stainings of ARP-T1 (red) and EHD4 (green). Nuclei are stained with Hoechst (blue). Scale bar, 10 µm.

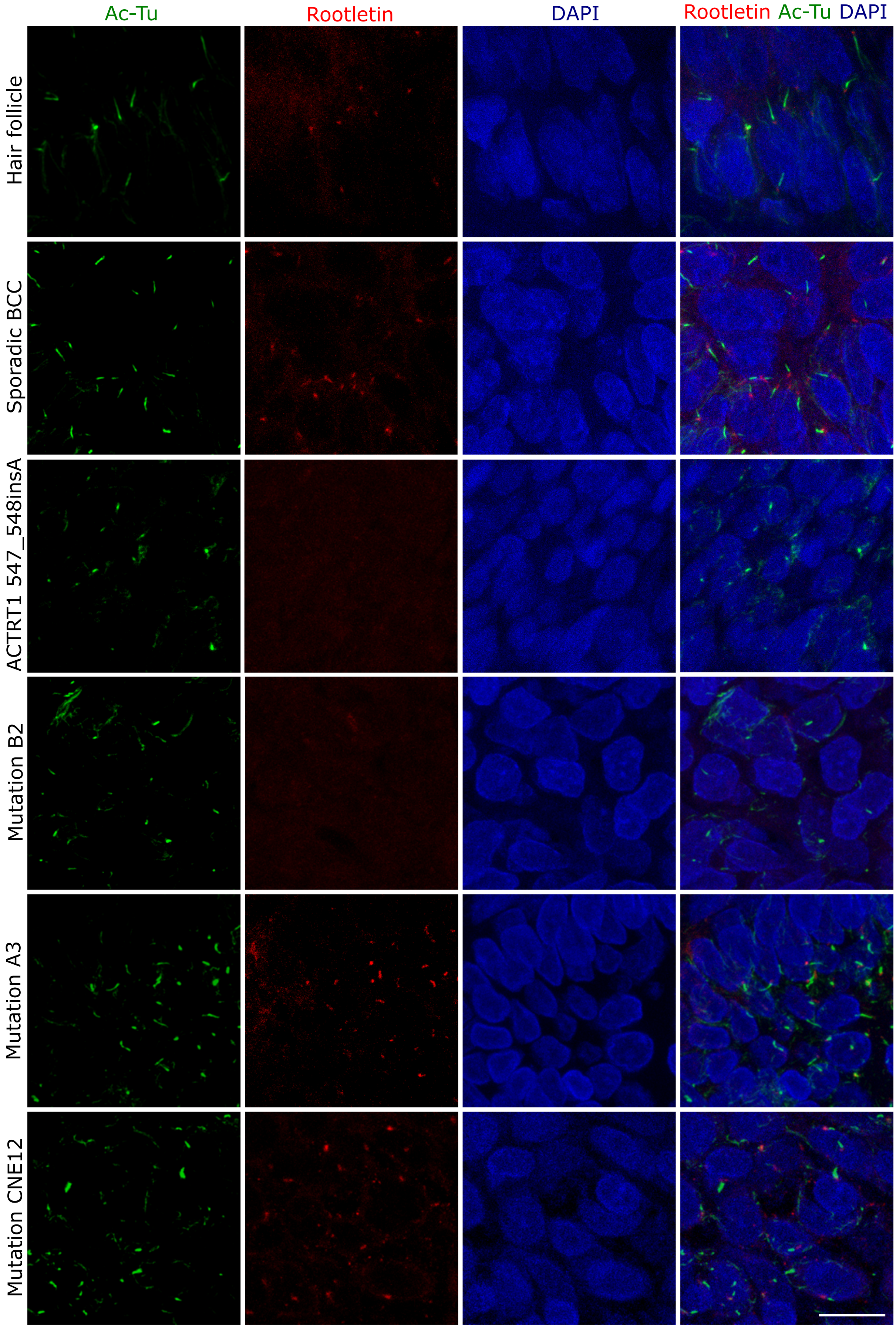

Supplementary Fig.2. Acetylated-tubulin and rootletin staining in patient samples. Representative immunofluorescence images with individual staining of acetylated-tubulin (green) and rootletin (red) in hair follicle, sporadic BCC and four BDCS (*ACTRT1 547_548insA*, Mutation B2, Mutation A3, Mutation CNE12). Cell nuclei are stained with DAPI (blue). Scale bar, 5 µm.

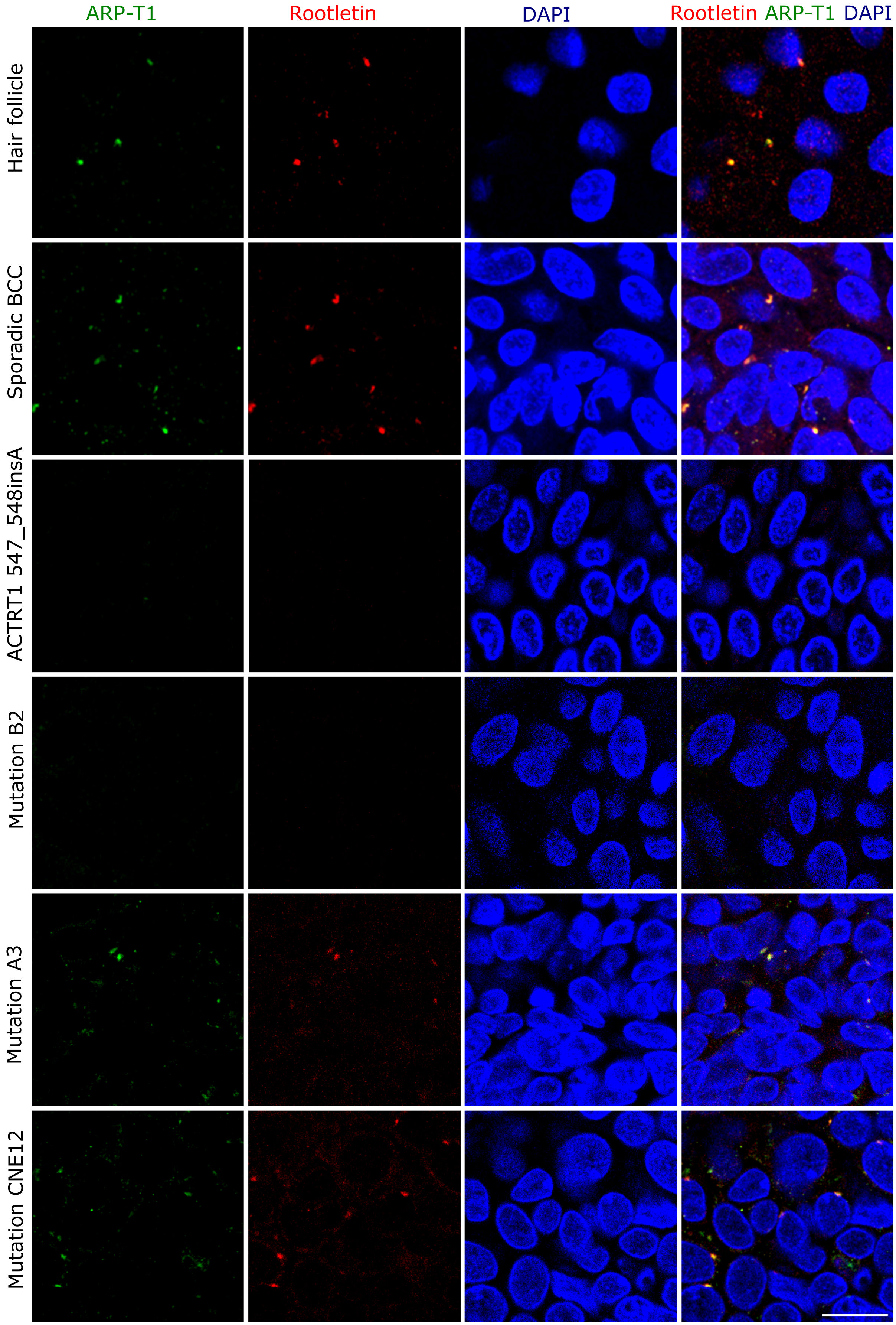

Supplementary Fig.3. ARP-T1 and rootletin staining in patient samples.
Representative immunofluorescence images of individual staining of ARP-T1 (green) and rootletin (red) in hair follicle, sporadic BCC and four BDCS (*ACTRT1 547_548insA*, Mutation B2, Mutation A3, Mutation CNE12). Cell nuclei are stained with DAPI (blue). Scale bar, 5 µm.

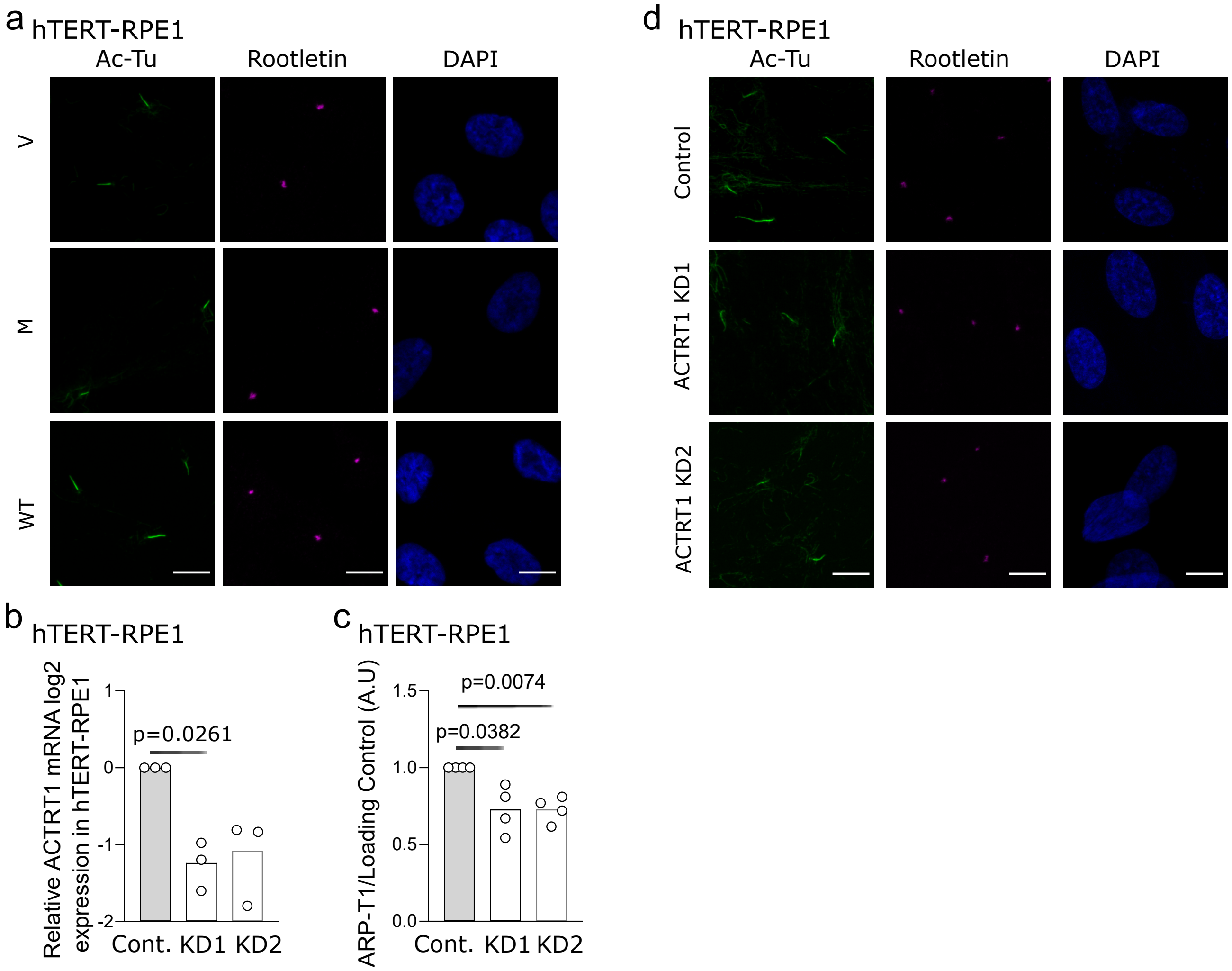

Supplementary Fig.4. ARP-T1 involvement in ciliogenesis in hTERT-RPE1 cells.

a, Individual immunofluorescence stainings of acetylated-tubulin (green) and rootletin (pink) in 48 h serum-starved hTERT-RPE1 cells expressing an empty vector (V), or *ACTRT1* mutant (M), or *ACTRT1* WT (WT). Cell nuclei are stained with DAPI (blue). Scale bar, 10 µm. **b**,**c**, Relative ACTRT1mRNA (**b**, N=3) and ARP-T1 (**c**, N=4) expression in control and ACTRT1KD hTERT-RPE1 cells. Data are presented as means of the fold change compared to the value of control cells. Each open circle represents one independent experiment. **d**, Individual immunofluorescence stainings of acetylated-tubulin (green) and rootletin (pink) in 48 h serum-starved control (Cont.) and ACTRT1 KD hTERT-RPE1 cells. Cell nuclei are stained with DAPI (blue). Scale bar, 10 µm.

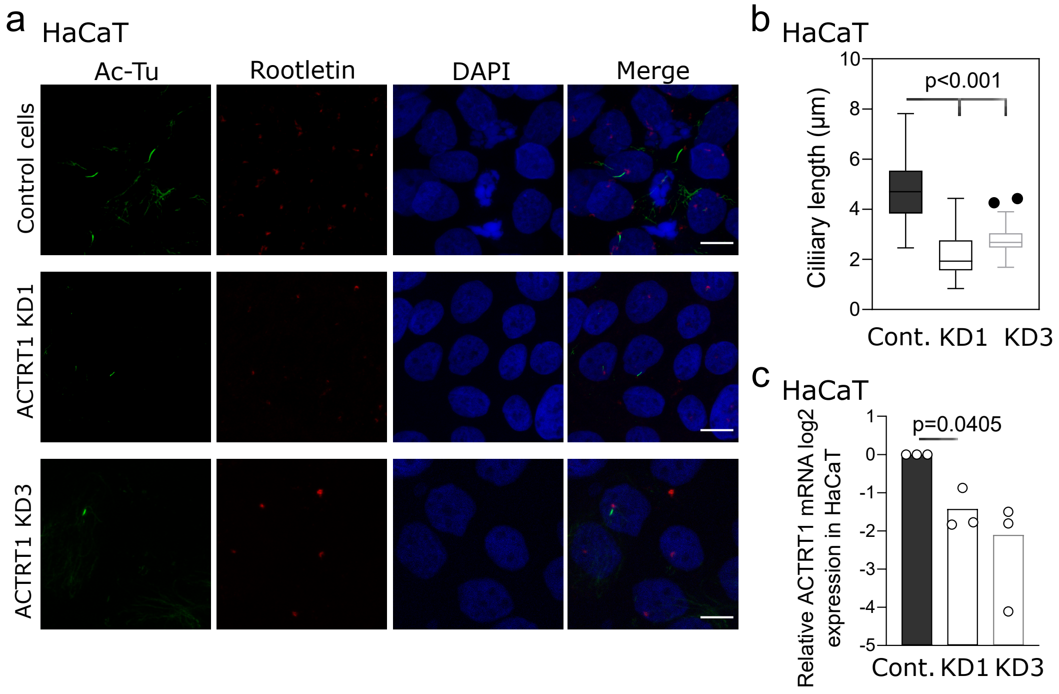

Supplementary Fig.5. ARP-T1 involvement in ciliogenesis in HaCaT cells.

a, Immunofluorescence stainings of acetylated-tubulin (green) and rootletin (red) in 7 days differentiated control and ACTRT1 KD HaCaT cells. Nuclei are stained with DAPI (blue). Scale bar, 10 μm. **b**,Quantification of ciliary length of **a**. Results are represented as Tukey box-plot. Black circles represent outliers. **c**, Relative ACTRT1 mRNA expression in control (Cont.) and ACTRT1 KD HaCaT cells. Data are presented as means of the fold change compared to the value of control cells. Each open circle represents one independent experiment (N=3).

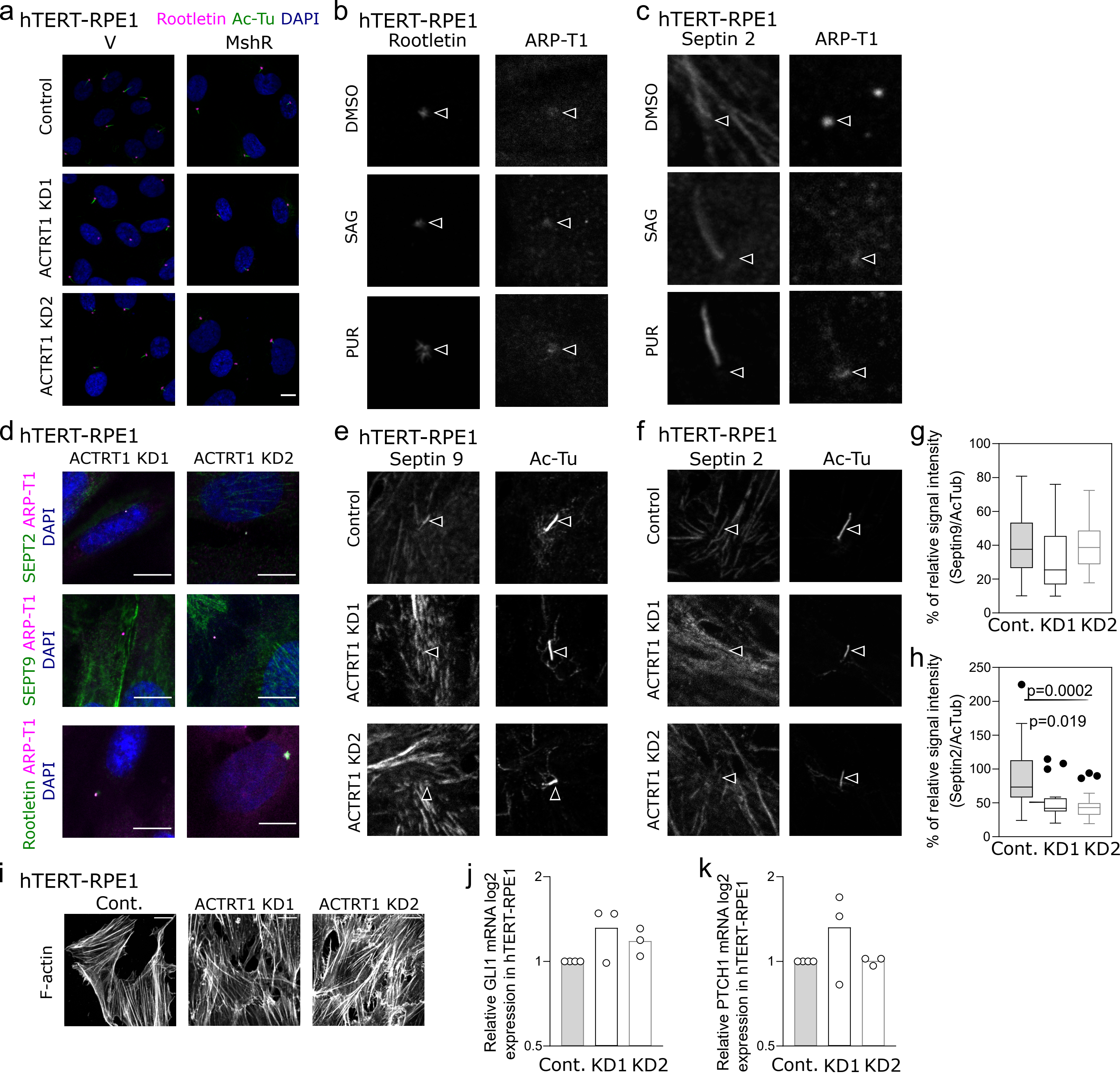

Supplementary Fig.6. ARP-T1 deficiency in hTERT-RPE1 cells.

a, Immunofluorescence stainings of acetylated-tubulin (Ac-Tu) (green) and rootletin (pink) in 48 h serum-starved hTERT-RPE1 cells expressing an empty vector (V) or *ACTRT1* mutant resistant shRNA (MshR). Cell nuclei are stained with DAPI (blue). Scale bar, 10 µm. **b**,**c**, Invididual grayscale staining of rootletin and ARP-T1 (**b**) and septin 2 and ARP-T1 (**c**) upon treatment with SAG or purmorphamine (PUR) in differentiated hTERT-RPE1 cells. The arrows show ARP-T1 localization. **d**, Immunofluorescence stainings of ARP-T1 (pink) and septin 2 (green, top) or septin 9 (green, middle) or rootletin (green, bottom) in 48 h serum-starved control (Cont.) and ACTRT1 KD hTERT-RPE1 cells. Cell nuclei are stained with DAPI (blue). Scale bar, 10 µm. **e**,**f**, Invididual grayscale staining of septin 9 and Ac-Tu (**e**) and septin 2 and Ac-Tu (**f**) in differentiated control (Cont.) and ACTRT1 KD hTERT-RPE1 cells. The arrows show Ac-Tu localization. **g**,**h**, Quantification of the signal intensity in **e** and **f**. Results are presented as Tukey box-plot. Black circles represent outliers (N=20-35). **i**, Invididual grayscale staining of actin in differentiated control (Cont.) and ACTRT1 KD hTERT-RPE1 cells. Scale bar, 10 µm **j**,**k**, Relative GLI1 and PTCH1 mRNA expression in control and ACTRT1 KD hTERT-RPE1 cells. Data are presented as means of the fold change compared to the value of control cells. Each open circle represents one independent experiment (N=3).

Supplementary Fig. 7. Uncropped immunoblots.
Full labelled images with ladder. Red rectangles show the cropped regions.

Fig 1b NHEK

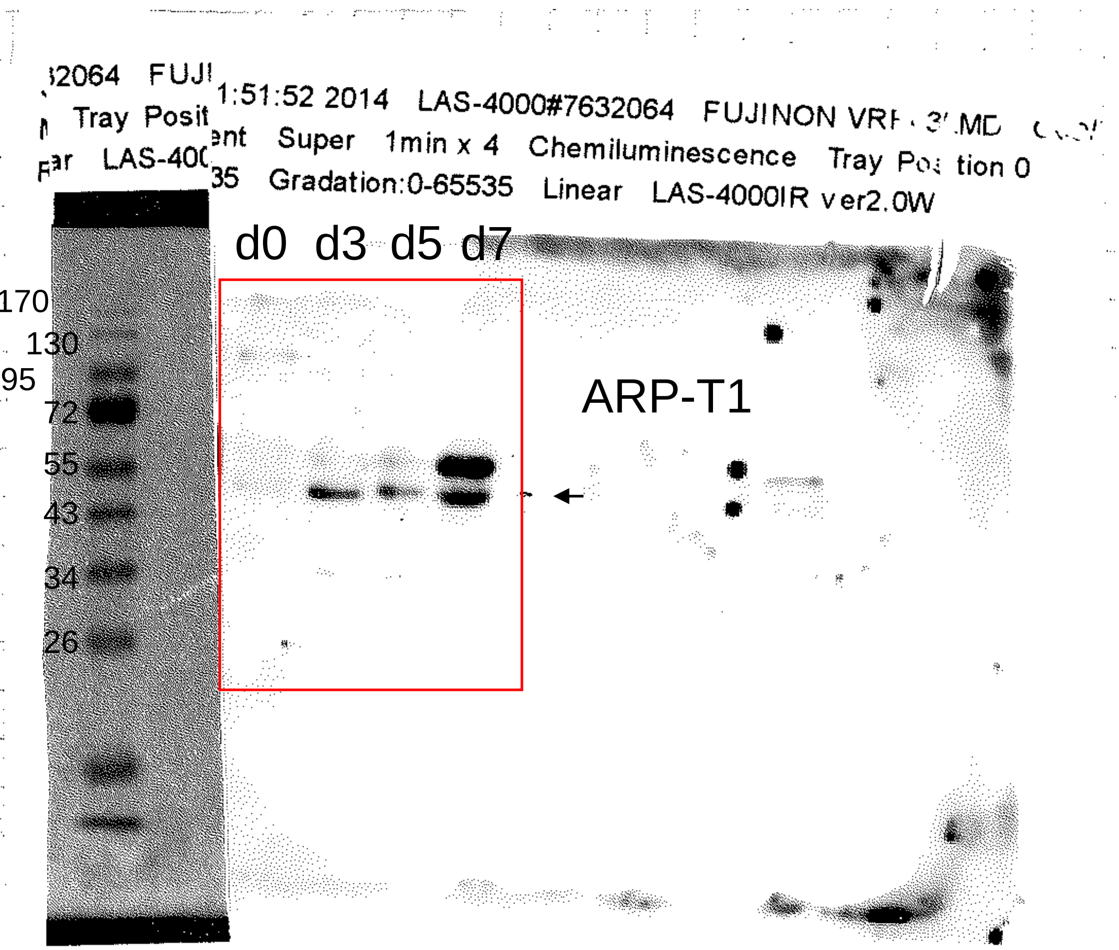

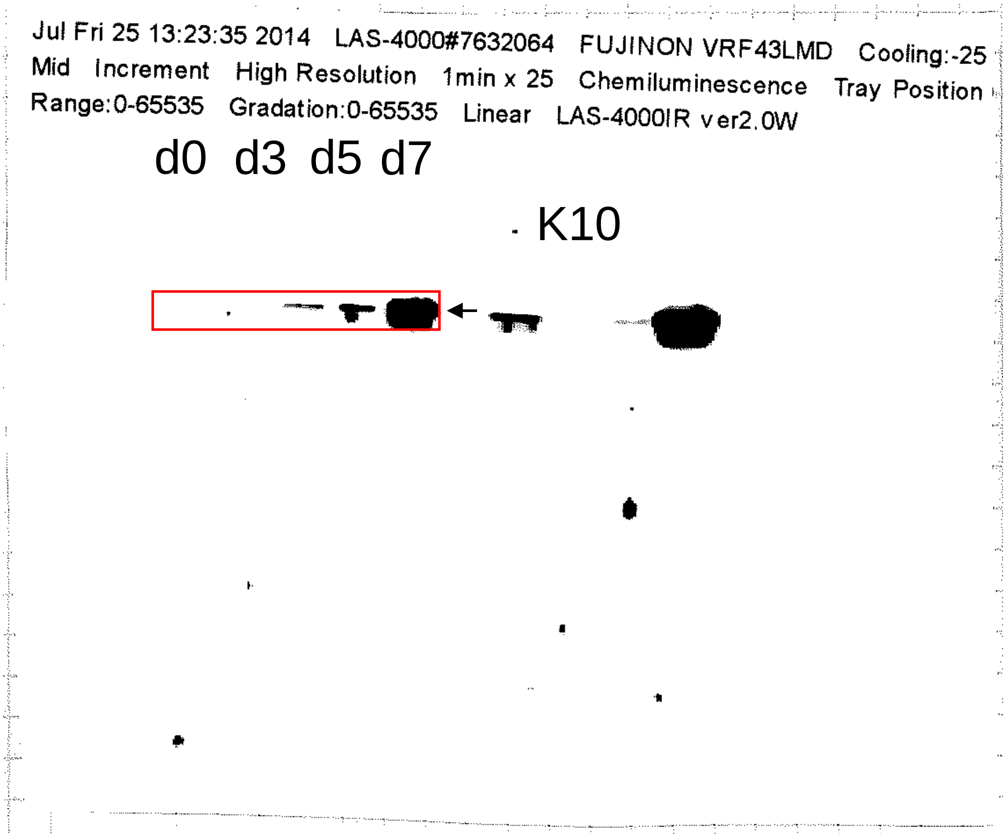

Fig 1b
NHEK

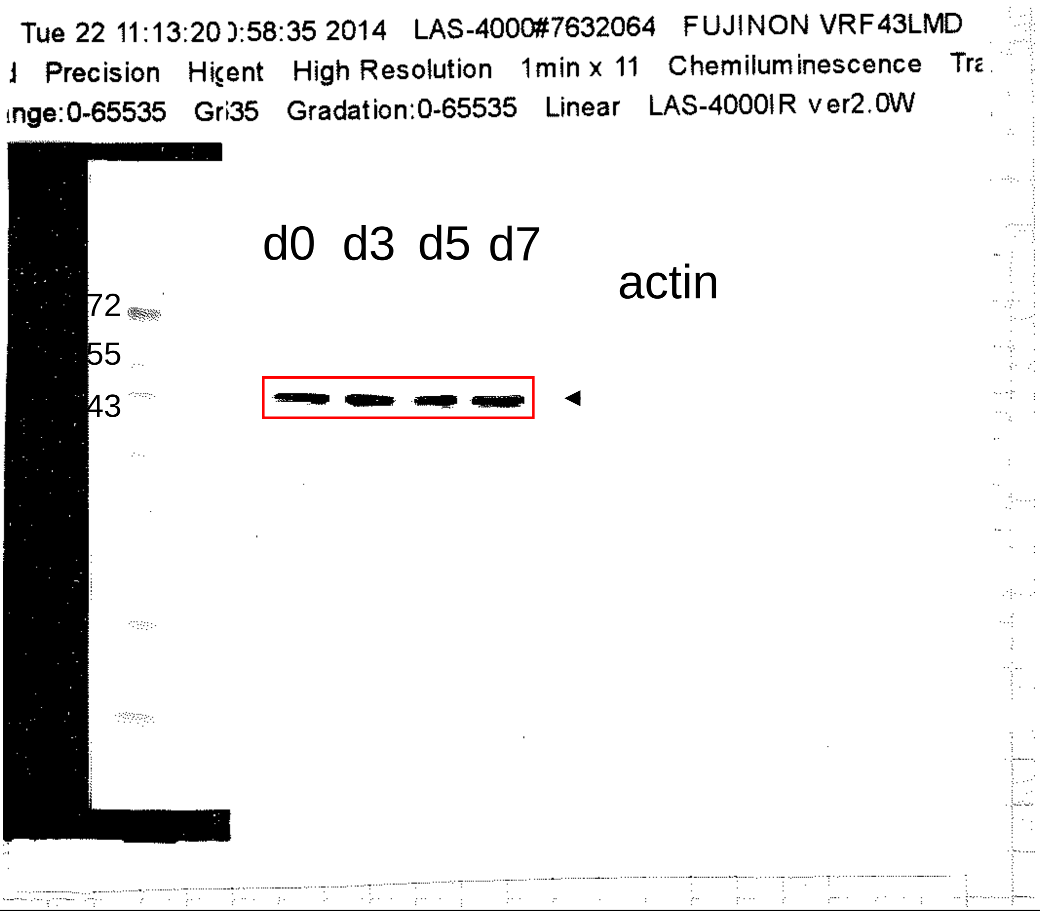

Fig 1d
HaCaT

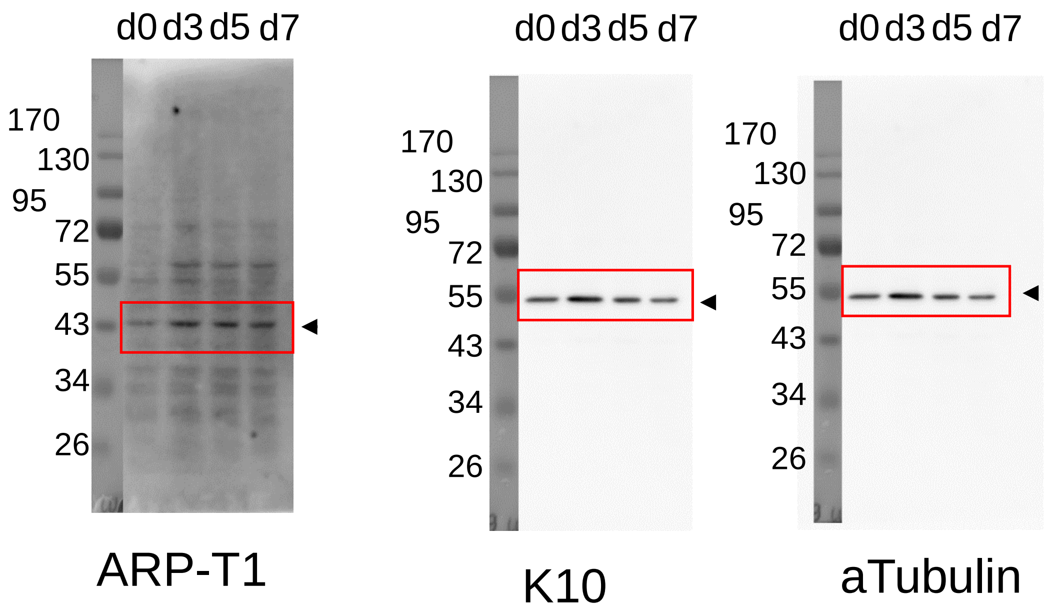

Fig 1f
ARPE19

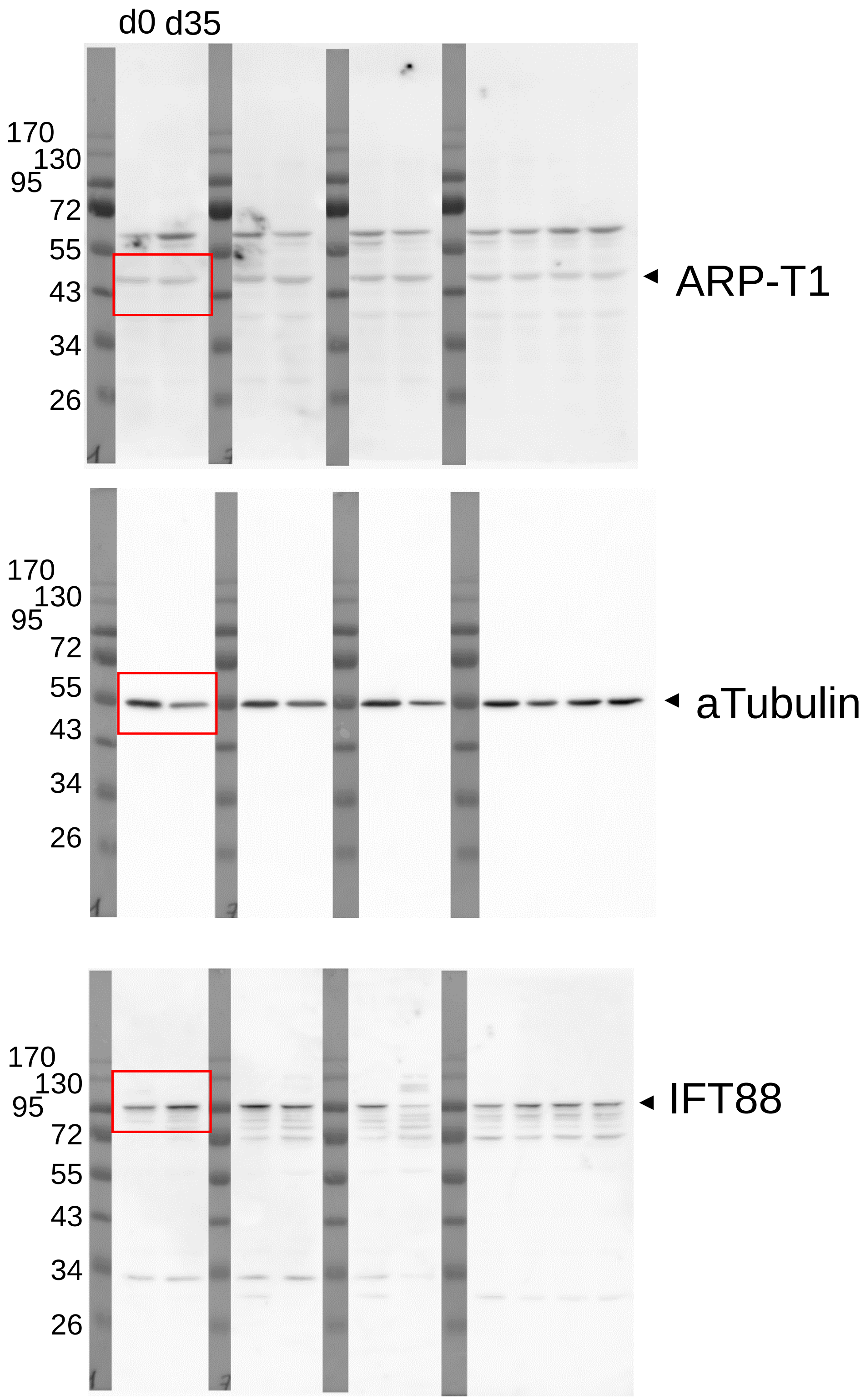

Fig 1h
hTERT RPE1

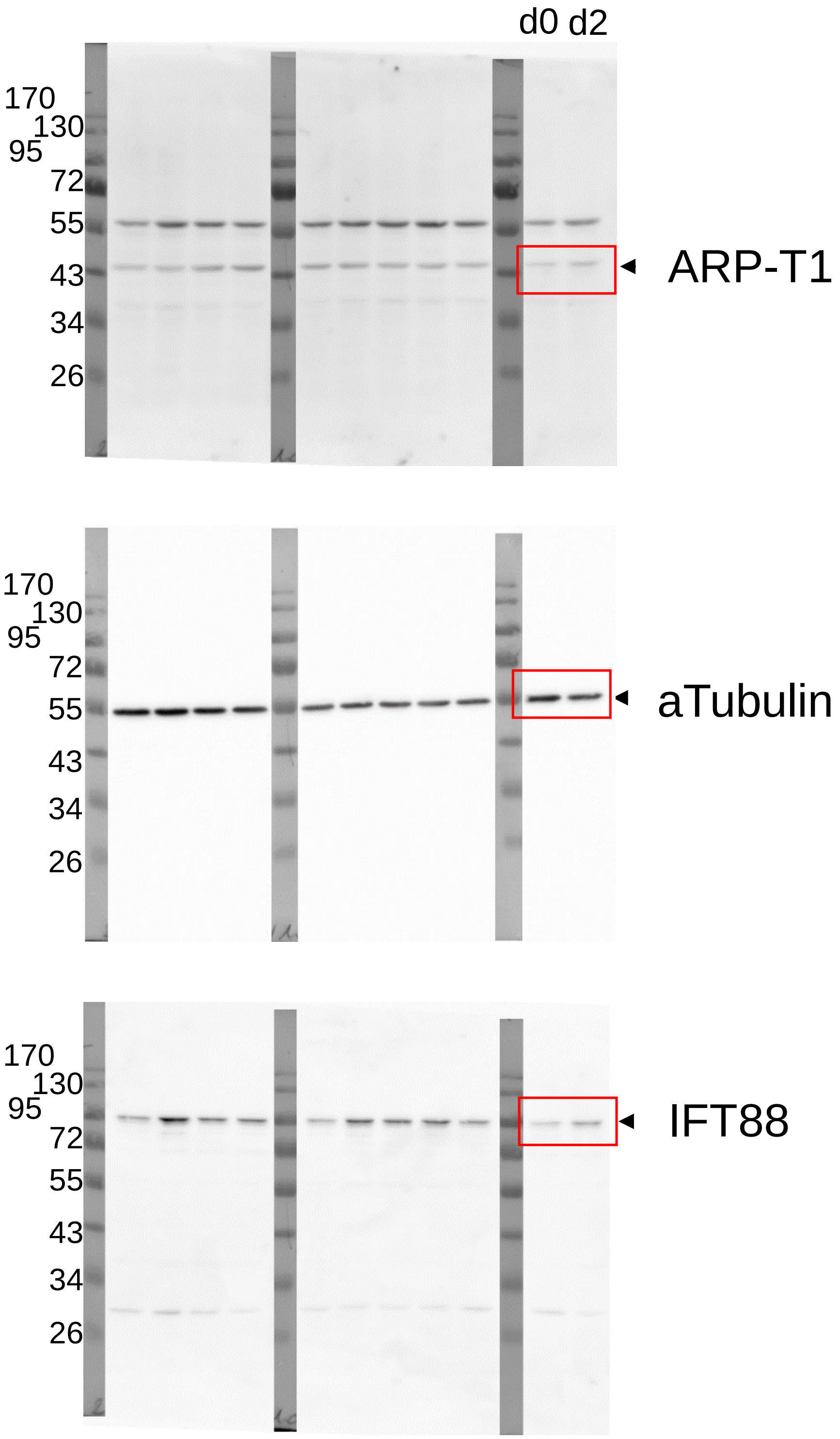

Fig 2a

hTERT RPE1, proliferating condition

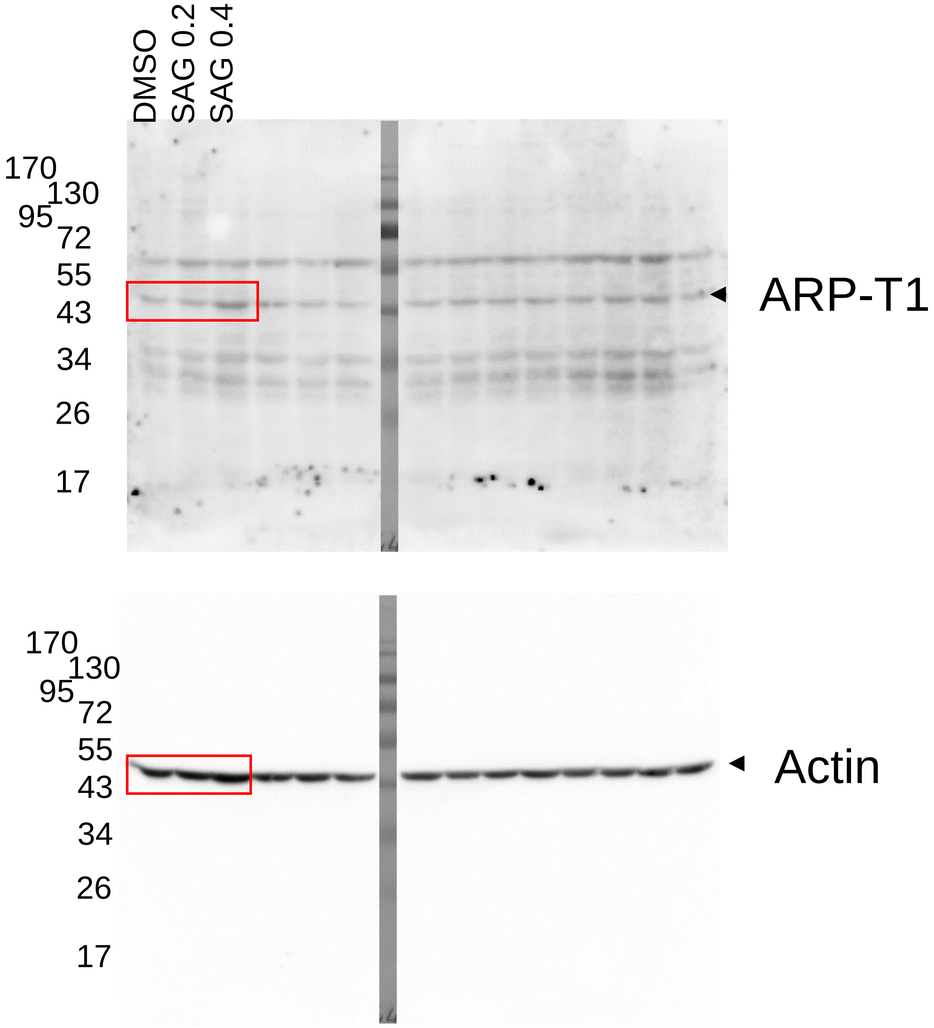

Fig 2b

hTERT RPE1, differentiating condition

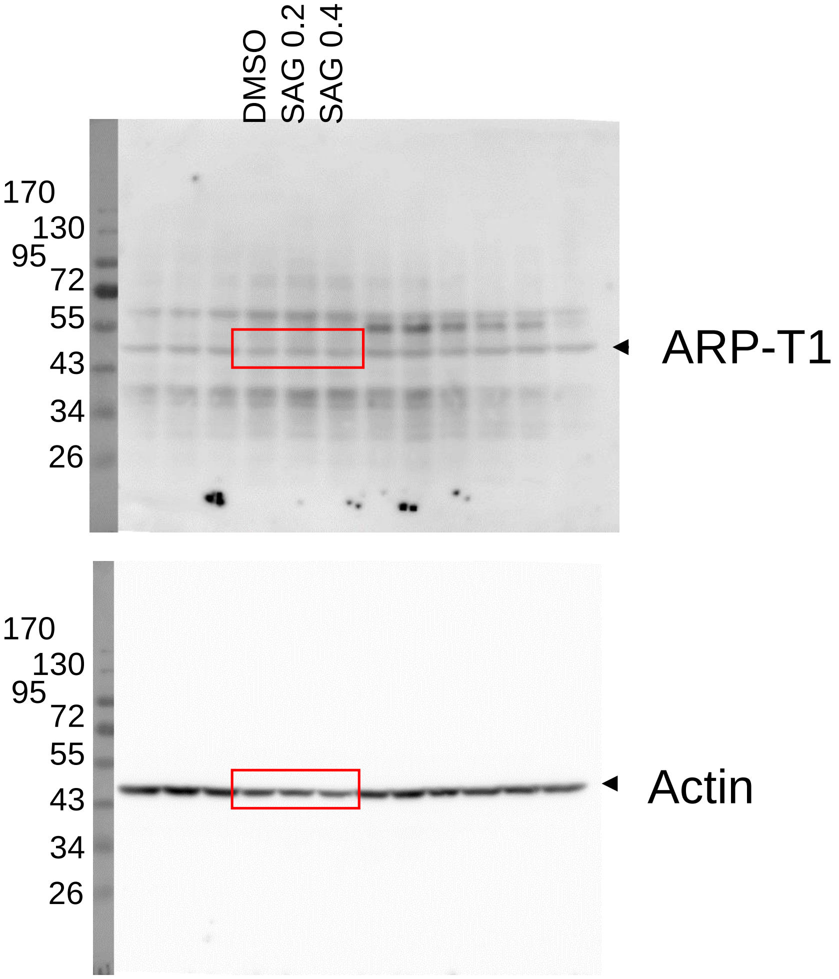

Fig 2c
hTERT RPE1, proliferating condition

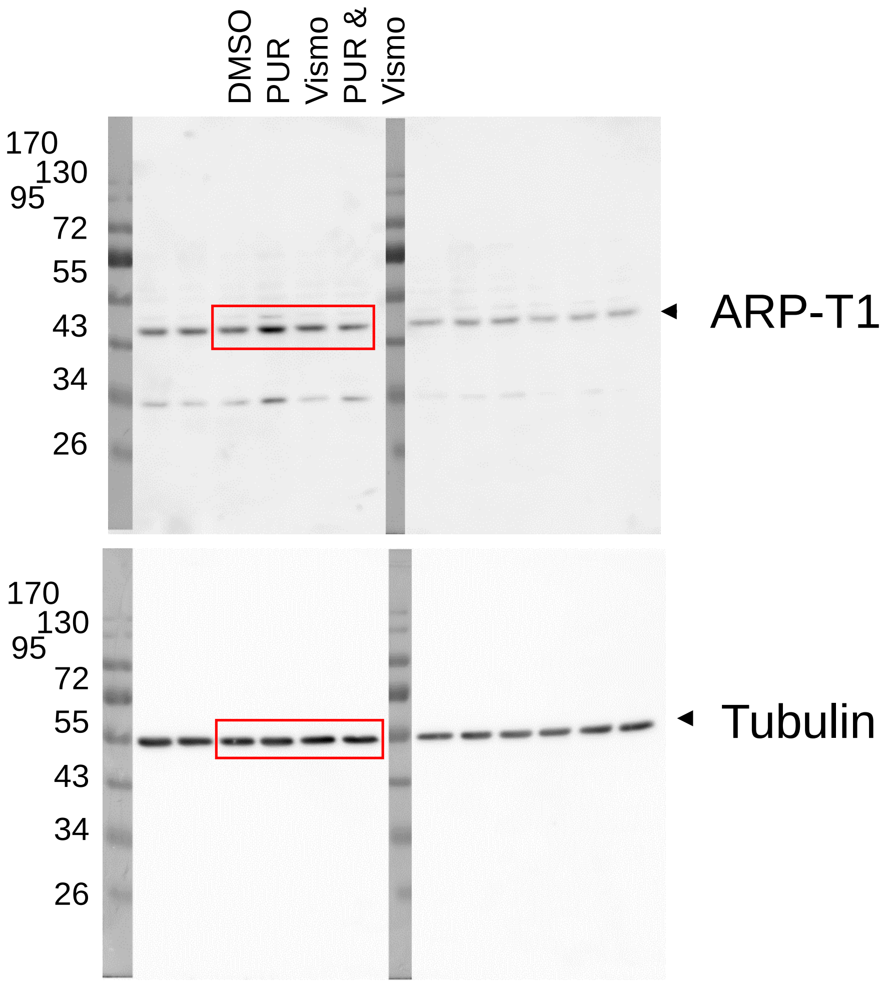

Fig 2j
hTERT RPE1

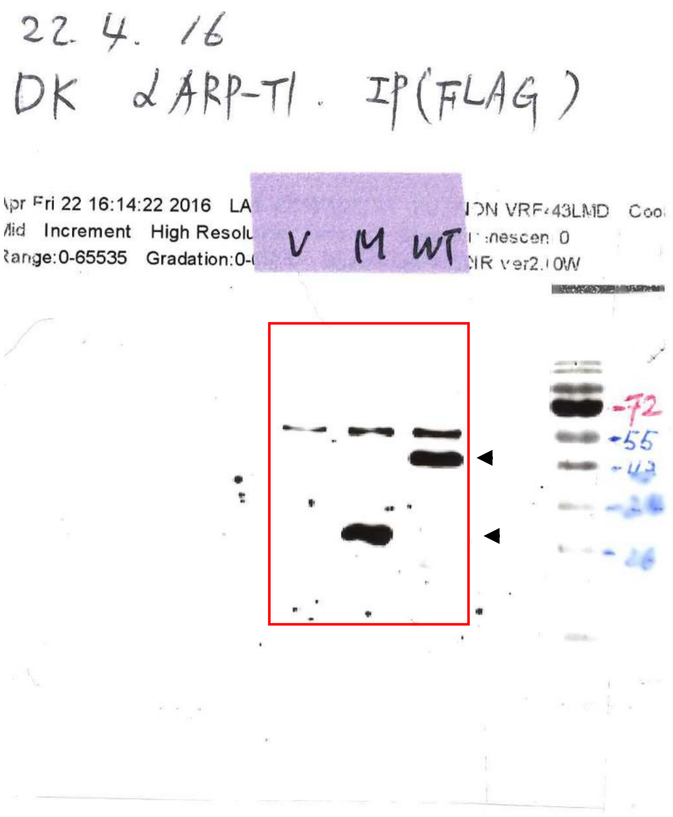

ARP-T1

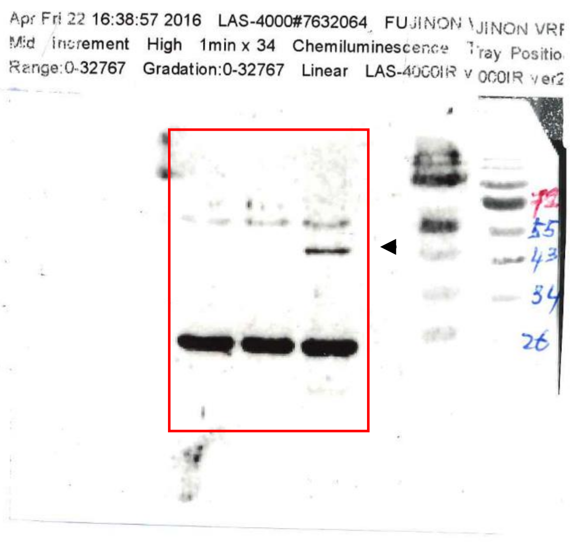

P-Ser PKC

Fig 3a
HeLa

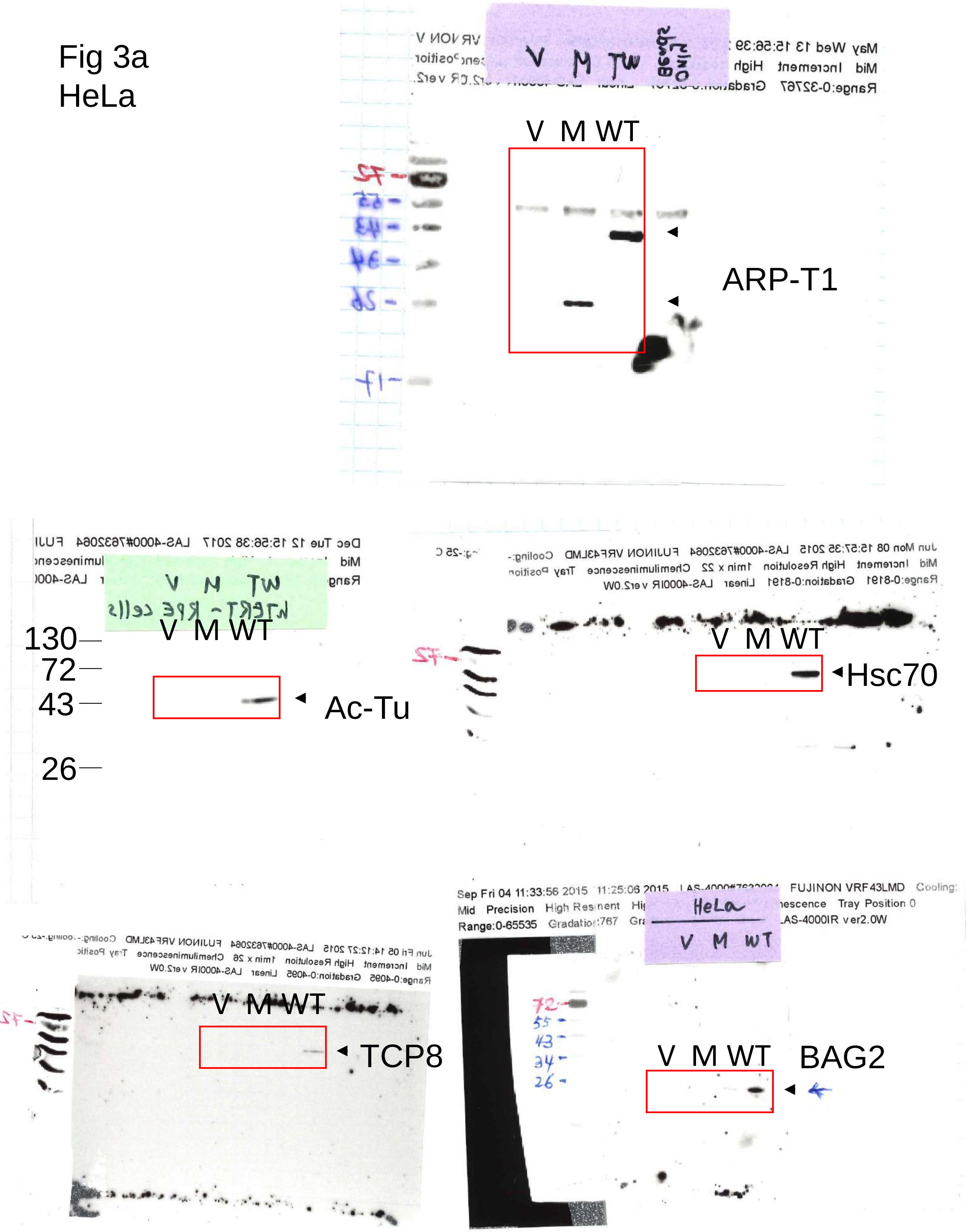
V M WT
ARP-T1
V M WT
130
72
43
26
Ac-Tu
V M WT
Hsc70
V M WT
TCP8
V M WT
BAG2
HeLa
Sep Fri 04 11:33:56 2015
Mid Precision High Res
Range:0-65535

Fig 3b
hTERT RPE1

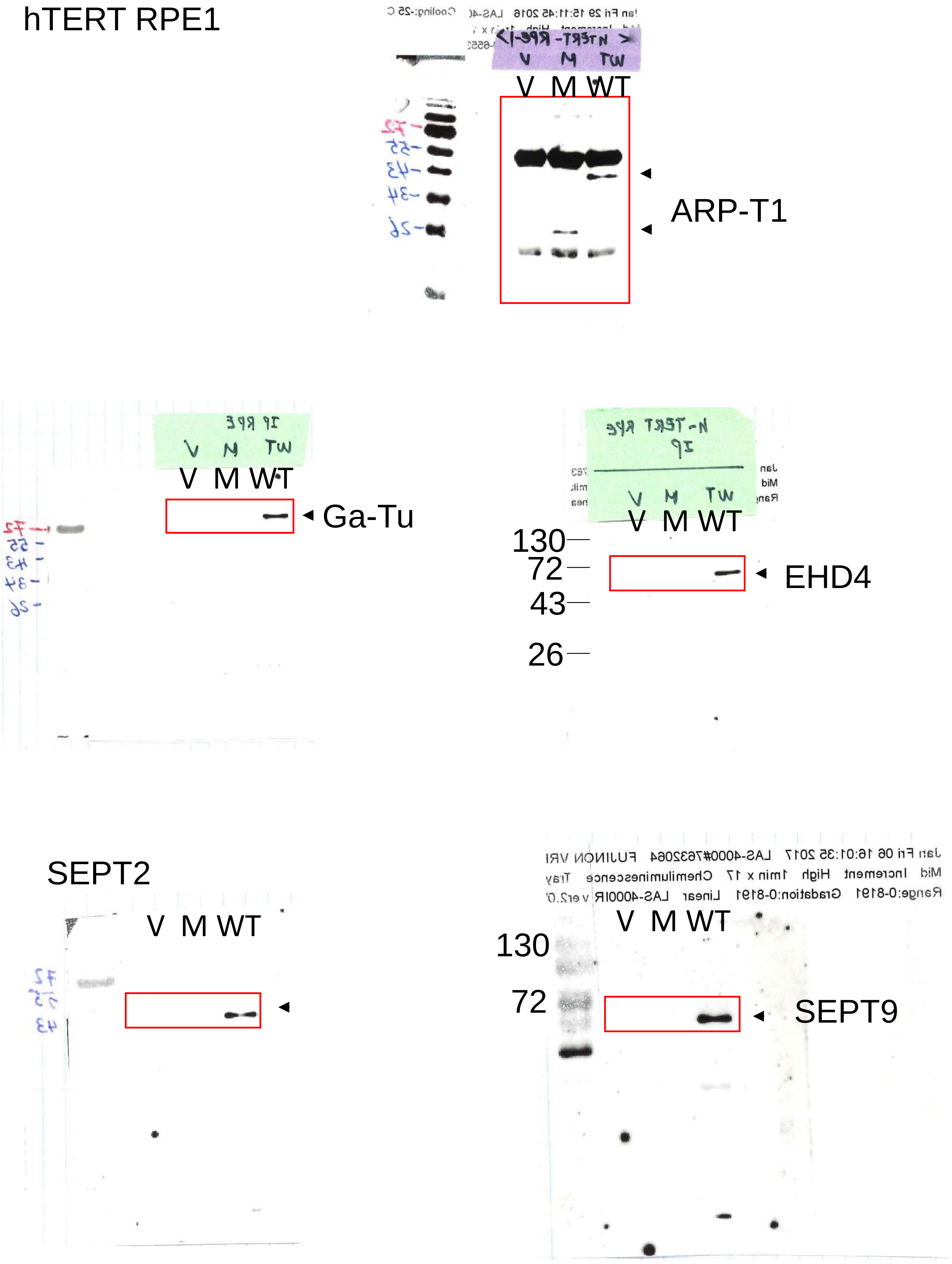

Fig 4k
hTERT RPE1

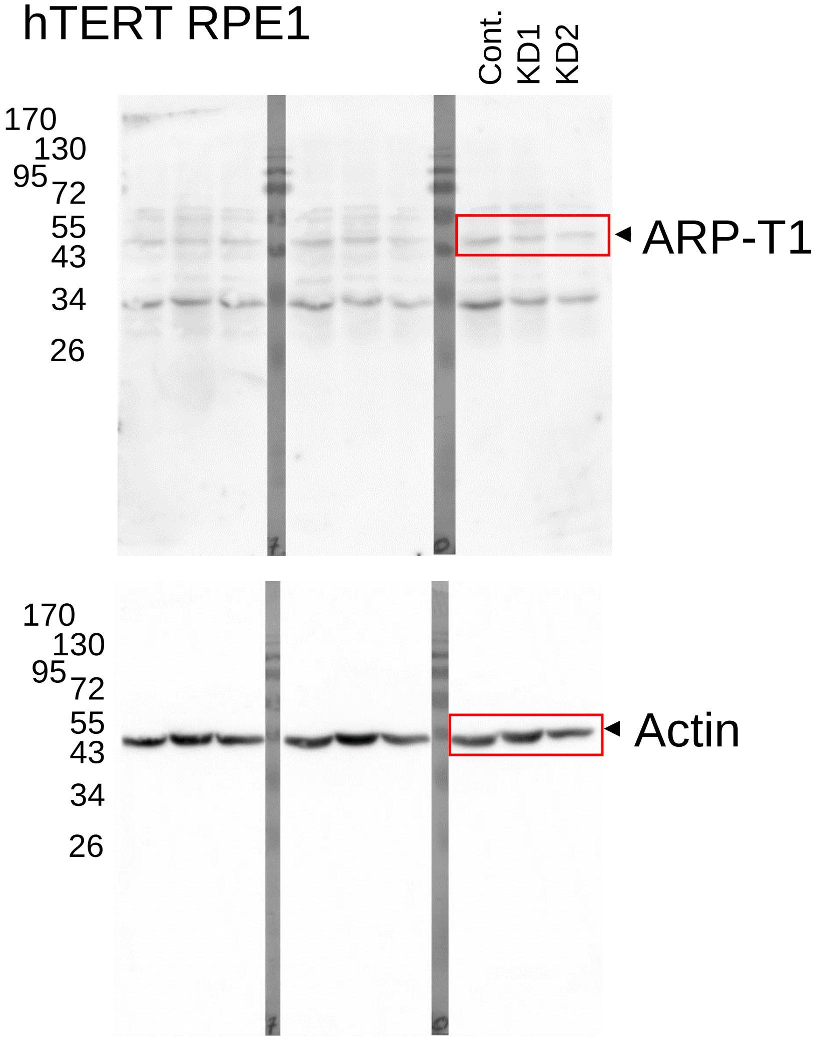

Supplementary Fig 1h
hTERT RPE1

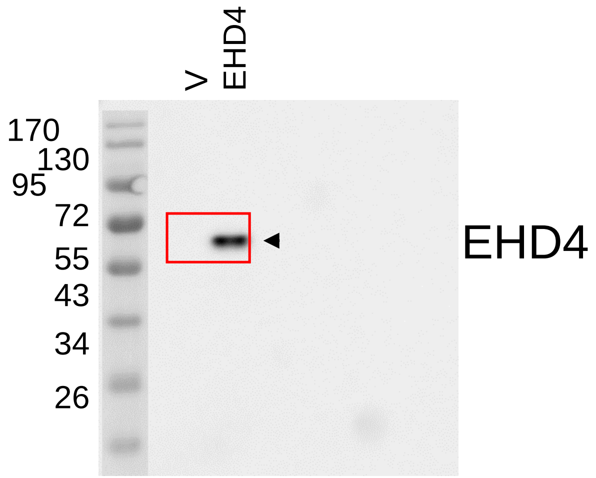

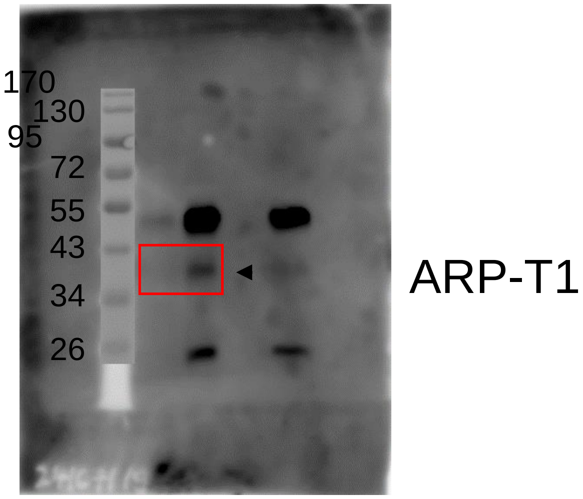

Pathway identifier	Pathway name	#Entities found	#Entities total	Entities ratio	Entities pValue	Entities FDR[a]
R-HSA-1430728	Metabolism	49	2135	1.91E-01	1.83E-03	9.14E-03
R-HSA-392499	Metabolism of proteins	56	2012	1.80E-01	3.06E-06	1.69E-04
**R-HSA-597592**	**Post-translational protein modification**	**32**	**1417**	**1.27E-01**	**1.65E-02**	**4.28E-02**
R-HSA-168249	Innate Immune System	31	1186	1.06E-01	2.32E-03	9.30E-03
R-HSA-1643685	Disease	27	1173	1.05E-01	2.20E-02	4.40E-02
**R-HSA-5653656**	**Vesicle-mediated transport**	**24**	**761**	**6.80E-02**	**6.64E-04**	**3.91E-03**
R-HSA-8953854	Metabolism of RNA	18	675	6.03E-02	1.66E-02	4.28E-02
**R-HSA-199991**	**Membrane Trafficking**	**21**	**635**	**5.68E-02**	**8.15E-04**	**4.08E-03**
R-HSA-1640170	Cell Cycle	17	622	5.56E-02	1.59E-02	4.28E-02
**R-HSA-422475**	**Axon guidance**	**20**	**558**	**4.99E-02**	**4.05E-04**	**2.43E-03**
R-HSA-168256	Immune System	169	2822	1.98E-01	9.33E-04	8.40E-03
R-HSA-392499	Metabolism of proteins	157	2354	1.65E-01	8.16E-06	1.72E-04
R-HSA-168249	Innate Immune System	86	1328	9.32E-02	0.00287	2.17E-02
R-HSA-1280215	Cytokine Signaling in Immune system	86	1261	8.85E-02	6.58E-04	5.92E-03
R-HSA-1280218	Adaptive Immune System	83	999	7.01E-02	9.83E-07	5.02E-05
**R-HSA-5653656**	**Vesicle-mediated transport**	**63**	**824**	**5.78E-02**	**2.21E-04**	**2.43E-03**
R-HSA-8953854	Metabolism of RNA	62	782	5.49E-02	9.45E-05	1.23E-03
**R-HSA-199991**	**Membrane Trafficking**	**59**	**665**	**4.67E-02**	**6.19E-06**	**1.42E-04**
R-HSA-8953897	Cellular responses to external stimuli	48	586	4.11E-02	2.89E-04	2.95E-03
**R-HSA-422475**	**Axon guidance**	**50**	**584**	**4.10E-02**	**7.68E-05**	**9.98E-04**

[a] FDR False Discovery Rate

Supplementary Table 1: Top10 of deregulated pathways in differentiated keratinocytes *ACTRT1* WT vs M (top) and hTERT-RPE1 *ACTRT1* WT vs M (bottom) analyzed with Reactome. Pathways in bold are linked to cilia and intracellular transport.

SUPPLEMENTARY METHODS

In situ proximity-mediated ligation assay (PLA)

hTERT-RPE1 cells were seeded on coverslips, fixed and permeabilized. Duolink in situ PLA kit with anti-rabbit PLUS probe and anti-mouse or anti-goat MINUS probe (Sigma-Aldrich) was used according to manufacturer`s instructions: blocking for 30 min in a 37°C humidified chamber, incubation with primary antibodies (gamma-tubulin (1:500, ab11316), ARP-T1 (1:100, SAB2103464), rootletin (1:50, sc-67824), EHD4 (1:200, ab83859, Abcam), septin 2 (1:100, HPA018481), septin 9 (1:100, HPA042564) for 2 h at RT or overnight at 4°C, hybridization with PLA PLUS and MINUS probes (1:5 dilution) for 1 h at 37°C, ligation, amplification and final washes. Nucleus was stained with DAPI for 2 min at RT. Coverslips were mounted onto slides and complex formation was examined with an inverted Zeiss LSM 700 microscope.

Transfection of EHD4 plasmid and reverse co-immunoprecipitation

hTERT-RPE1 cells were transfected using ESCORT IV Transfection Reagent (L 3287, Sigma-Aldrich), with pcDNA6.V5 and pcDNA6.V5 hEHD4 WT (kind gift from Dr. Markus Plomann, unpublished construct), according to manufacturer`s instructions: first, for 2 x 10e5 cells, 1.5 ug DNA and 2 ug ESCORT IV were diluted separately in OPTIMEM (Gibco), then mixed, and incubated 15 min at RT. After two washes in PBS, the mix was drop-wised on the cells, which were then incubated for 6 h at 37°C. The transfection was stopped by the addition of 20% FBS-DMEM/F12 medium and the cells were grown for 48 h at 37°C.

A co-immunoprocipitation was performed on the cells, using anti-V5 Agarose Affinity Gel antibody produced in mouse (A7345, MERCK) following the protocol described in the main methods section. EHD4 precipitation was confirmed using EHD4 antibody (1:500, ab153892 Abcam) and co-precipitated ARP-T1 was analyzed using ARP-T1 antiserum (1:2000, GP-SH6).

F-actin immunofluorescence

The immunofluorescence protocol from the main Methods was used. Staining was performed with phalloidin-alexa fluor 546 (1:200, A22283, Invitrogen) 1h at RT and DAPI for 5 min at RT. Coverslips were mounted onto slides and actin filaments were analyzed with an inverted Zeiss LSM 700 microscope.
