# Supplementary material for: ARP-T1-associated Bazex–Dupré–Christol syndrome is an inherited basal cell cancer with ciliary defects characteristic of ciliopathies
Source: Commun Biol. 2021 May 10;4:544. doi: 10.1038/s42003-021-02054-9 (PMC8110579; doi:10.1038/s42003-021-02054-9)
Supplement: Supplementary file 2 — Description of Additional Supplementary Files [file 42003_2021_2054_MOESM2_ESM.pdf]

Description of Additional Supplementary Files

File name: Supplementary Data 1

Description: All numerical data supporting the graphs of the main figures.
