# Supplementary material for: ARP-T1-associated Bazex–Dupré–Christol syndrome is an inherited basal cell cancer with ciliary defects characteristic of ciliopathies
Source: Commun Biol. 2021 May 10;4:544. doi: 10.1038/s42003-021-02054-9 (PMC8110579; doi:10.1038/s42003-021-02054-9)
Supplement: Supplementary file 4 — Reporting Summary [file 42003_2021_2054_MOESM4_ESM.pdf]

nature research

Corresponding author(s): Daniel Hohl

Last updated by author(s): Mar 24, 2021

Reporting Summary

Nature Research wishes to improve the reproducibility of the work that we publish. This form provides structure for consistency and transparency in reporting. For further information on Nature Research policies, see Authors & Referees and the Editorial Policy Checklist.

Statistics

For all statistical analyses, confirm that the following items are present in the figure legend, table legend, main text, or Methods section.

n/a	Confirmed	
☐	☒	The exact sample size (*n*) for each experimental group/condition, given as a discrete number and unit of measurement
☐	☒	A statement on whether measurements were taken from distinct samples or whether the same sample was measured repeatedly
☐	☒	The statistical test(s) used AND whether they are one- or two-sided<br>*Only common tests should be described solely by name; describe more complex techniques in the Methods section.*
☒	☐	A description of all covariates tested
☐	☒	A description of any assumptions or corrections, such as tests of normality and adjustment for multiple comparisons
☐	☒	A full description of the statistical parameters including central tendency (e.g. means) or other basic estimates (e.g. regression coefficient) AND variation (e.g. standard deviation) or associated estimates of uncertainty (e.g. confidence intervals)
☒	☐	For null hypothesis testing, the test statistic (e.g. *F*, *t*, *r*) with confidence intervals, effect sizes, degrees of freedom and *P* value noted<br>*Give P values as exact values whenever suitable.*
☒	☐	For Bayesian analysis, information on the choice of priors and Markov chain Monte Carlo settings
☒	☐	For hierarchical and complex designs, identification of the appropriate level for tests and full reporting of outcomes
☒	☐	Estimates of effect sizes (e.g. Cohen's *d*, Pearson's *r*), indicating how they were calculated

Our web collection on statistics for biologists contains articles on many of the points above.

Software and code

Policy information about availability of computer code

Data collection	Immunofluoresence images: Zen 2010 v6.0 (Zeiss); Wester blot: ImageQuant LAS4000 v1.3 (GE Healthcare); RT-qPCR: StepOne software v2.3 (Applied Biosystems); Mass spectrometry: Xcalibur software (Thermo Fisher)
Data analysis	Excel 2016 (Microsoft), ImageJ v1.52 (NIH), Prism v8 (GraphPad), Mascot 2.5 (Matrix Science), Scaffold software (version 4.4, Proteome Software Inc.)

For manuscripts utilizing custom algorithms or software that are central to the research but not yet described in published literature, software must be made available to editors/reviewers. We strongly encourage code deposition in a community repository (e.g. GitHub). See the Nature Research guidelines for submitting code & software for further information.

Data

Policy information about availability of data

All manuscripts must include a data availability statement. This statement should provide the following information, where applicable:

- Accession codes, unique identifiers, or web links for publicly available datasets
- A list of figures that have associated raw data
- A description of any restrictions on data availability

The mass spectrometry datasets (Table1 and S1) generated and analysed during the current study are available in the ProteomeXchange reposity, with identifier PXD016557. They can be accessed via https://www.ebi.ac.uk/pride/archive/login , using the login: reviewer62648@ebi.ac.uk ; and password: e2T3DqJd
After acceptation of the manuscript, the datasets will be publicly available.
Original immunoblots and immunofluorescence data are accessible on Zenodo repository with the dataset identifier 10.5281/zenodo.4570029 (all versions: 10.5281/zenodo.3666278).

Field-specific reporting

Please select the one below that is the best fit for your research. If you are not sure, read the appropriate sections before making your selection.

☒ Life sciences ☐ Behavioural & social sciences ☐ Ecological, evolutionary & environmental sciences

For a reference copy of the document with all sections, see nature.com/documents/nr-reporting-summary-flat.pdf

Life sciences study design

All studies must disclose on these points even when the disclosure is negative.

Sample size	For RNA and protein expression one to three samples were analyzed each time. For confocal images, 20 to 50 cells were analyzed for each sample in one experiment.
Data exclusions	No data were excluded.
Replication	All experiments were performed at least three times.
Randomization	No randomization was needed in this study.
Blinding	No blinding was needed in this study.

Reporting for specific materials, systems and methods

We require information from authors about some types of materials, experimental systems and methods used in many studies. Here, indicate whether each material, system or method listed is relevant to your study. If you are not sure if a list item applies to your research, read the appropriate section before selecting a response.

Materials & experimental systems

n/a	Involved in the study	
☐	☒	Antibodies
☐	☒	Eukaryotic cell lines
☒	☐	Palaeontology
☒	☐	Animals and other organisms
☐	☒	Human research participants
☒	☐	Clinical data

Methods

n/a	Involved in the study	
☒	☐	ChIP-seq
☐	☐	Flow cytometry
☒	☐	MRI-based neuroimaging

Antibodies

Antibodies used	For western-blot: anti-ARP-T1 (1:1000, SAB1408334, Sigma-Aldrich / 1:2000, GP-SH6, Progen), anti-keratin 10 (1:200, MS-611-P0, Thermo Scientific),), anti-IFT88 (1:1000, 13967-1-AP, Proteintech, UK), anti-actin (1:2000, A2066, Sigma-Aldrich) and anti-alpha-tubulin (1:5000, T9026, Sigma-Aldrich), anti-mouse (1:5000, NA931V, GE Healthcare UK Limited), anti-rabbit (1:5000, NA934V, GE), anti-guinea pig (1:5000, ab97155, Abcam). For co-immunoprecipitation: anti-ARP-T1 (1:2000, Progen GP-SH6), anti-acetylated-tubulin (1:1000, T6793, Sigma-Aldrich), anti-TCP8 / TCP1 theta (1:500, PA5-30403, Thermo Scientific), anti-HCS70 (1:200, PA5-27337, Thermo Scientific), anti-BAG2 (1:100, PA5-30922, Thermo Scientific), anti-gamma-tubulin (1:500, #ab11316, Abcam), anti-EDH4 (1:1000, Dr.Plomann's lab), anti-septin2 (1:2000, HPA018481, Sigma-Aldrich), anti-septin9 (1:2000, HPA042564, Sigma-Aldrich). For immunofluorescence: anti-ARP-T1 (1:100, #SAB2103464; 1:200, Progen GP-SH6), anti-acetylated-tubulin (1:1000, T6793), anti-rootletin (1:50, sc-67824, Santa-Cruz; 1:200, NBP1-80820, Novus), anti-gamma-tubulin (1:500, ab11316, Abcam), anti-EDH4 (1:200, Dr.Plomann's lab), anti-septin2 (1:100, HPA018481, Sigma-Aldrich), anti-septin9 (1:2000, HPA042564, Sigma-Aldrich), secondary antibodies (1:500, A11008, A11001, A21467, A11035, A11003, A11060, Invitrogen; 1:100, BA-9500, Vector Laboratories; 1:200, RPN1233V, GE). Actin filaments were stained with phalloidin-alexa fluor 546 (1:200, A22283, Invitrogen).
Validation	All antibodies were optimised and used following the manufactures' instructions.

Eukaryotic cell lines

Policy information about cell lines

Cell line source(s)	NHEK were established in our laboratory. HaCaT cells were bougth at Invitrogen (Basel, Switzerland), ARPE-19 and hTERT-RPE1 at ATCC (Manassas, VA).

Authentication	None of the cell lines used were authentified in our lab.
Mycoplasma contamination	Cell lines were not tested for mycoplasma contamination, as kept in culture for less than 10 passages
Commonly misidentified lines (See ICLAC register)	*Name any commonly misidentified cell lines used in the study and provide a rationale for their use.*

Human research participants

Policy information about studies involving human research participants

Population characteristics	Patient samples were already used in our previous publication (Bal et al. 2017 Nature Medicine). All the details are therefore published.
Recruitment	*Describe how participants were recruited. Outline any potential self-selection bias or other biases that may be present and how these are likely to impact results.*
Ethics oversight	*Identify the organization(s) that approved the study protocol.*

Note that full information on the approval of the study protocol must also be provided in the manuscript.

Flow Cytometry

Plots

Confirm that:

- [ ] The axis labels state the marker and fluorochrome used (e.g. CD4-FITC).
- [ ] The axis scales are clearly visible. Include numbers along axes only for bottom left plot of group (a 'group' is an analysis of identical markers).
- [ ] All plots are contour plots with outliers or pseudocolor plots.
- [x] A numerical value for number of cells or percentage (with statistics) is provided.

Methodology

Sample preparation	*Describe the sample preparation, detailing the biological source of the cells and any tissue processing steps used.*
Instrument	*Identify the instrument used for data collection, specifying make and model number.*
Software	*Describe the software used to collect and analyze the flow cytometry data. For custom code that has been deposited into a community repository, provide accession details.*
Cell population abundance	*Describe the abundance of the relevant cell populations within post-sort fractions, providing details on the purity of the samples and how it was determined.*
Gating strategy	*Describe the gating strategy used for all relevant experiments, specifying the preliminary FSC/SSC gates of the starting cell population, indicating where boundaries between "positive" and "negative" staining cell populations are defined.*

- [ ] Tick this box to confirm that a figure exemplifying the gating strategy is provided in the Supplementary Information.
